# P-633. Implementation and Uptake of Nirsevimab within Nemours Children’s Health Delaware Healthcare System

**DOI:** 10.1093/ofid/ofae631.830

**Published:** 2025-01-29

**Authors:** Sara Mann, Matt Mason, Neil Rellosa, Karen Ravin

**Affiliations:** Nemour's Children's Health, Wilmington, Delaware; Nemours Children's Hospital, Delaware, Wilmington, Delaware; Nemour's Children's Health, Wilmington, Delaware; Nemour's Children's Health, Wilmington, Delaware

## Abstract

**Background:**

Nirsevimab, the first FDA approved monoclonal antibody to protect against Respiratory Syncytial Virus (RSV), became available for the 2023-2024 RSV season. Distribution of nirsevimab was affected by cost and a supply shortage, limiting overall uptake. Nemour’s Children’s Hospital in Delaware (NCH) implemented several strategic measures to promote immunization despite these barriers, including educational series and periodic communication of regional supply chain status. The purpose of this project was to analyze characteristics of patients who received nirsevimab at NCH and corresponding trends over the 2023-2024 season.

**Figure:**
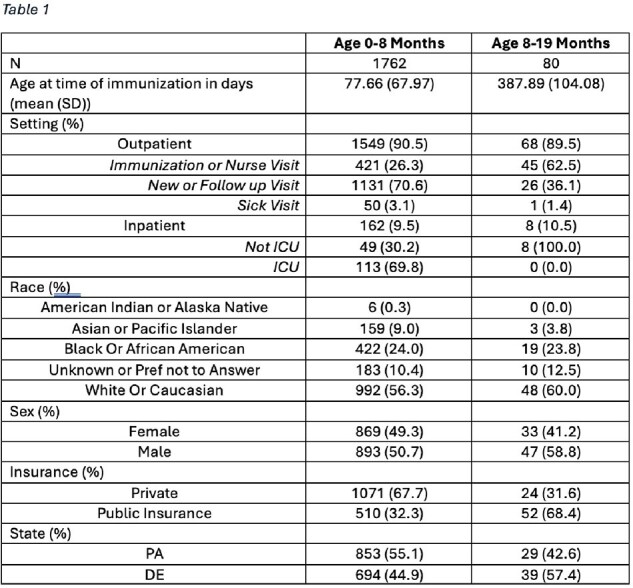

**Methods:**

We report descriptive summaries of patients who received nirsevimab at NCH from October 2023 to March 2024. Variables analyzed included age, timing of immunization, clinical site and setting, race, sex, insurance status, and state where the patient received the immunization (PA or DE). We compared the distributions of these characteristics by vaccination month using chi-square tests.

**Figure:**
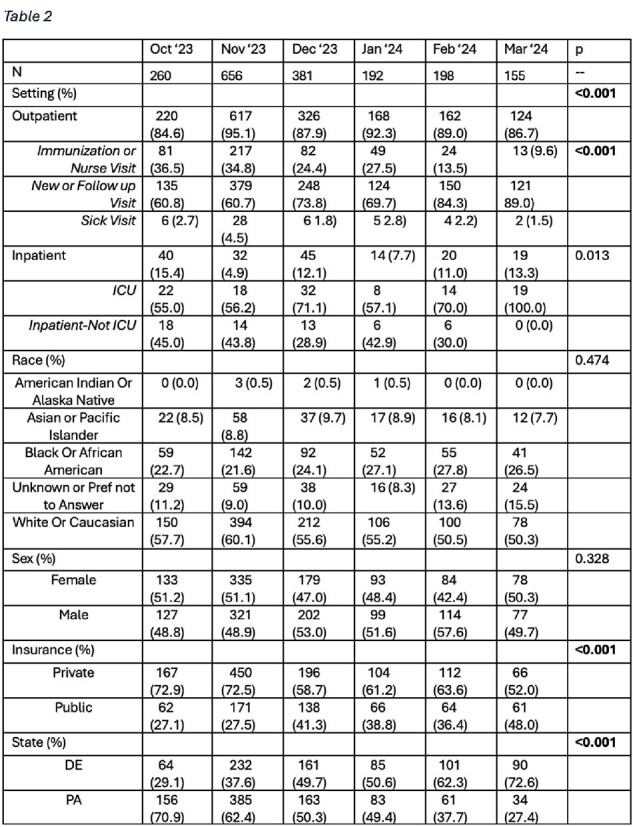

**Results:**

1,842 infants received nirsevimab, most of which were under 8 months of age (95.7%) at time of immunization (Table 1). Nirsevimab uptake varied by month (Figure 1), with most patients receiving the immunization in November and December. The distribution of immunizations across visit type (outpatient/inpatient, p< 0.001), insurance type (public/private, p< 0.001), and state (DE/PA) varied significantly across months. The distributions of patient-reported sex and race did not vary significantly by month (Table 2).

**Figure:**
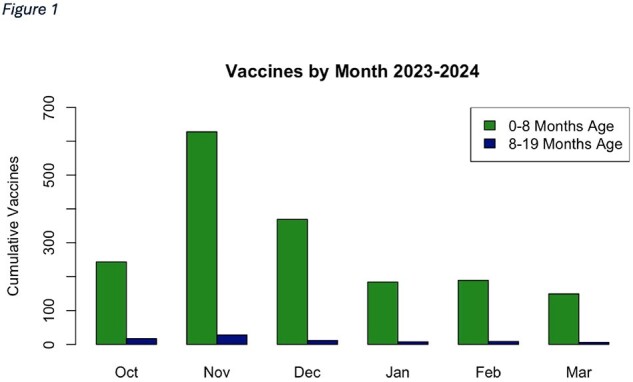

**Conclusion:**

Unpredictable supplies of nirsevimab and varying immunization strategies among clinic sites affected distribution and uptake among patients. Some clinics were faced with prioritizing high-risk populations or were restricted by patient insurance policies. These challenges resulted in differing populations having access to nirsevimab based on time of year. Further efforts can focus on unifying immunization promotion techniques and identifying ways to ensure equitable distribution to our target populations.

**Disclosures:**

**All Authors**: No reported disclosures

